# Prioritizing Suggestive Candidate Genes in Migraine: An Opinion

**DOI:** 10.3389/fneur.2022.910366

**Published:** 2022-06-15

**Authors:** Simona Denise Frederiksen

**Affiliations:** Independent Researcher, Calgary, AB, Canada

**Keywords:** GWAS, bioinformatics, gene prioritization, headache, migraine, causation, pathophysiology, membrane potential

## Introduction

The search for the underlying causes of migraine has been ongoing for decades, with genome-wide association studies (GWASs) enabling the discovery of common single nucleotide polymorphisms (SNPs) associated with this disorder, along with suggestive candidate genes (examples include PHACTR1, TRPM8 and PRDM16) (1–3). Suggestive candidate genes have predominantly been selected based on their genomic location and on expert knowledge. The term “*suggestive candidate gene*” reveals the level of evidence of the finding (i.e., suggesting a link between a gene and condition) thereby indicating that validation is necessary.

Gene prioritization, especially relying on computationally-intensive multi-omics analyses [e.g., weighing score based on evidence source (4)], has been used to help identify candidate genes truly associated with a condition (5–7). Gene prioritization is conducted to rank “*genes according to their likelihood of being associated with the disease”* and thus researchers can distinguish between credible and non-credible suggestive candidate genes, and thus select the most credible genes to further study (8). Meta-analyses of GWASs have also helped to confirm findings (2, 3). Even though some SNP-condition associations are non-reproducible, it is not enough evidence to rule out those findings. As we know, many common conditions are multifactorial in nature and the genetic architecture and regulatory networks differ between individual patients (9, 10).

Despite of the initial methodologies used to identify suggestive candidate genes being similar across migraine GWASs, the use of downstream gene prioritization varies. This may in part be explained by the continuous advancements of bioinformatics tools over time, but may also be explained by a lack of defined systematic gene prioritization efforts, particularly focused on causality. Adding an additional gene prioritization step in future GWASs to further prioritize identified suggestive candidate genes may (i) reduce the reporting of false positives, and (ii) enhance our etiological understanding of the disorder. As the number of suggestive candidate genes increases together with the number of publications, there is a growing need for valid gene prioritization (11). Here, the current and potential future state of gene prioritization in migraine GWASs will be discussed.

## Gene Prioritization in Migraine

Depending on the study objective, gene prioritization might help to answer the question: “*What is the likelihood of the suggestive candidate genes truly causing common migraine?”* Yet, here it is important to keep in mind that evidence points to a multifactorial etiology of common migraine (12), and that the causes of common migraine are largely unknown.

### Further Validation of Suggestive Candidate Genes Needed

GWASs have provided some clues about the migraine etiology, particularly at the SNP level. One limitation of this approach is the uncertainty of causality. For instance, the genotyped SNPs found to be significantly associated with migraine might be in linkage disequilibrium (LD) with the causal variants, rather than being causal themselves, and the LD structure might contain numerous genes (13, 14). Researchers have sought to find out how these SNPs may be associated with the disorder, and have frequently looked into whether those SNPs are located in coding or non-coding regions. If located in a non-coding region, suggestive candidate genes have primarily been identified focusing on the genes located nearest to the SNPs or on functionally-relevant genes in the proximate genomic region of the SNPs. It has however been found that “*only about one-third of causal genes are the nearest gene to the GWAS hit”* (13), and the implication of non-coding variants is rarely studied. So, even though the GWAS methodology itself is hypothesis-free, the identification of suggestive candidate genes has predominantly been hypothesis-driven. Findings from GWAS in migraine have been discussed by van den Maagdenberg and colleagues (15).

When examining existing migraine GWASs, suggestive candidate genes have primarily been identified by (i) examining genes in proximate genomic region (2, 3, 16–21), (ii) reporting the genes for coding SNPs (2, 3, 19, 20, 22, 23), (iii) reporting nearest gene (21, 22), and (iv) using LD analysis outputs for guidance (2, 16–19, 24). These methodologies cannot be used to infer causality, and selection of suggestive candidate genes in the proximate genomic regions of SNPs of interest is generally based on expert knowledge (and today's knowledge). Therefore, GWAS findings may be biased toward the perspectives held by those experts. For example, in addition to migraine being described as a neurovascular disorder, several other theories have been proposed throughout the years. Recently, researchers have started to describe migraine as a purely neurological condition (e.g., with “*primarily neuronal origin with the vascular manifestations”*) (25). Other theories have arisen throughout the past decade where researchers present migraine as a neuro-glio-vascular disorder (26) or dysfunctional neurolimbic pain network (27).

To account for some of these limitations, additional gene prioritization has been conducted in some migraine GWASs. Examples of applied downstream gene prioritization methodologies include tissue-based gene expression analysis (3), and expression quantitative trait locus (eQTL) analysis using human control tissues [e.g., umbilical cords (16), cerebellum and frontal cortex (3), thyroid and brain (17)]. Despite of the use of some advanced tools to prioritize suggestive candidate genes in migraine GWASs, there is still a gap in gene prioritization efforts that need to be addressed (e.g., causality is not thoroughly examined). The existence of this gap can in part be explained by the difficulty in obtaining relevant omics data of diseased tissue, especially for neurological conditions.

### Additional Gene Prioritization Step in Future GWASs

Due to the emergence of advanced bioinformatics tools, gene prioritization in GWASs can be taken a step further. This opportunity is important to consider as the combination of GWAS and eQTL does not inform us about whether “*gene expression and the trait are affected by the same underlying causal variant”* as stated by Zhu and colleagues (28). Causality cannot be inferred. Referring to the disease-associated loci, Cano-Gamez and Trynka state that “*it is unclear which genes they regulate”* (29).

There are several other reasons why filtering of the list of suggestive candidate genes is important, including (i) evidence sources such as the GWAS catalog (30, 31) are used in downstream bioinformatics analysis to examine potential involvement of genes in disease and (ii) researchers want to reveal how the genetic background of an individual influences their biological functions and disease susceptibility. If the cause(s) of a disorder is known, health professionals can provide more targeted treatment instead of just trying to manage symptoms. Importantly, applying our knowledge about genetic causes of familial/monogenic migraine may help us separate signal from noise among the GWAS findings (as causality in these cases have been established), and thus examine the clinical relevance of suggestive candidate genes in common migraine.

Currently, we know of the following monogenic forms of migraine: Familial hemiplegic migraine type 1 (FHM1; mutations in the calcium channel gene CACNA1A), type 2 (FHM2; mutations in the sodium/potassium-transporting ATPase gene ATP1A2) and type 3 (FHM3; mutations in the sodium channel gene SCN1A) (32, 33). Those genes all seem to affect neurotransmission, susceptibility for cortical spreading depression and cognitive function (34–42). Among families with migraine, mutations in several other genes, such as KCNK18 (potassium channel gene), ATXN1 (chromatin-binding factor gene) and CACNA1B (calcium channel gene), have been found (32, 43, 44). These six genes are involved in regulation of membrane potential (GO:0042391), based on ToppGene [a candidate gene prioritization tool] phenotype and functional annotations (45).

The first step toward conducting additional gene prioritization in future GWASs is to understand each component of a gene prioritization tool. A gene prioritization tool “*represents a unique combination of evidence sources, prioritization strategy and input requirements”*, as defined by Zolotareva and Kleine (46). Testing data, training data and evidence sources are used as inputs. Training data (genes used to prioritize) has previously been created based on established genes underlying familial forms of a disease, for example for Alzheimer's disease (46). To obtain training data, a list of genes previously linked to migraine (e.g., causative rather than susceptible) can be created based on the biomedical literature (46–48). Information stored in databases such as ClinVar (focus on genomic variation in human health) (49) and OMIM (based on reviews of the biomedical literature by experts) (50, 51) can also be utilized to enhance the decision process.

When using gene prioritization tools like ToppGene (45), ToppNet (45) or pBRIT (52), the user also needs to define the testing data (genes to prioritize). To obtain testing data, identified suggestive candidate genes in migraine GWAS can be used. Alternatively, the complete list of suggestive candidate genes can be identified through the NHGRI-EBI GWAS catalog (i.e., a curated collection of all published human GWAS) and corresponding R package gwasrapidd (30, 31). For some tools, the user can adjust training parameters (or use default settings). If using ToppGene, this includes choosing evidence sources/features (e.g., the Gene Ontology (GO) resource to explore gene functions (53), and PubMed to explore the biomedical literature). Overall, evidence sources (together with computational approaches) have been used to estimate gene similarity/proximity focused on the testing and training data (46).

**Figure 1:**
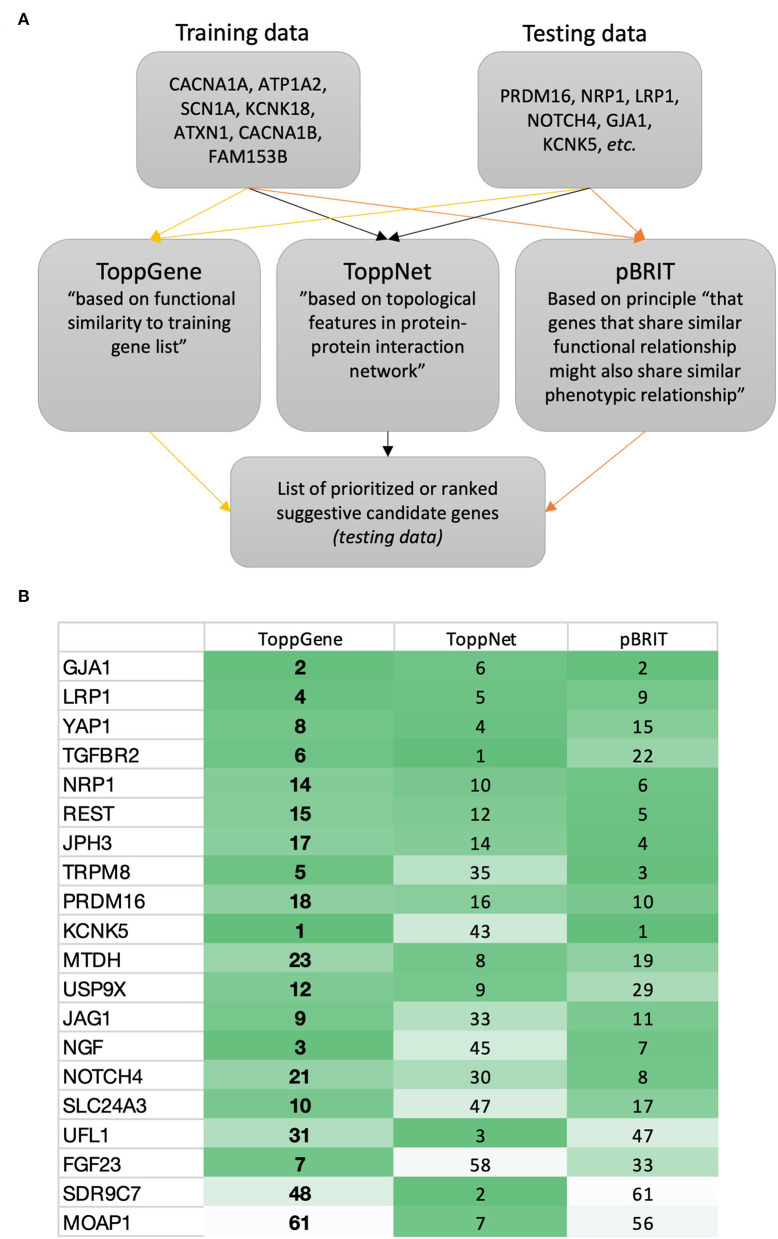
Proposed gene prioritization for future GWASs. **(A)** Suggested gene prioritization workflow. According to the described approach (i.e., creating training data focused on known familial migraine genes), the gene prioritization strategy will seek to prioritize suggestive candidate genes in relation to the genes forming the training data. This prioritization may be based on features such as similarity (e.g., focused on genetic sequence, involvement in biological pathways, or accompanying phenotypes) and/or proximity (e.g., in PPI network or focused on gene location and linkage) (46). Other tools then those described here exist (46, 53, 54). **(B)** Example of ToppGene (45), ToppNet (45), and pBRIT (52) outputs showing a selection of ranked suggestive candidate genes from migraine GWASs. Online resources: https://toppgene.cchmc.org/; http://143.169.238.105/pbrit/.

This proposed additional gene prioritization step is visualized in Figure 1A.

### Gene Prioritization Efforts in Existing GWASs

Use of established gene prioritization tools can help us to more confidently predict whether a suggestive candidate gene is credible or not or, more likely, to uncover how credible a suggestive candidate gene might be (i.e., ranking by score). This may help us to facilitate the selection of genes that are most likely to be associated with the migraine, beyond the capabilities of expression data.

The suggestive candidate genes GJA1 and KCNK5 (2) (rarely reported in migraine GWASs) ranked in the top 2 based on ToppGene and pBRIT outputs (Figure 1B; being mindful that databases continuously get updated). Both genes are involved in regulation of membrane potential (GO:0042391) as are the majority of genes known to cause familial/monogenic migraine. Yet, Gormley and colleagues stated that “*loci identified to date do not support the idea of common variants in ion channel genes being strong susceptibility components in prevalent forms of migraine”* (2). However, recent migraine GWAS findings point in another direction. Hautakangas and colleagues found a risk variant in CACNA1A that seemed to be specific for migraine with aura, and stated that “*CACNA1A seems involved in both monogenic and polygenic forms of migraine”* (55).

This indicates that the proposed gene prioritization step (Figure 1A) is likely to be beneficial for future migraine GWASs.

## Discussion

Here, use of gene prioritization to score and rank suggestive candidate genes in migraine was discussed. In some migraine GWASs, expression data from control human tissues (difficulty in obtaining diseased human brain tissues) have been used to prioritize suggestive candidate genes. Even if diseased human brain tissues were used, such analysis is not able to infer causality of the genetic variants. Hence, the overall goal with this opinion piece is to advance the conversation about gene prioritization in GWAS, presented from the perspective of migraine.

As we already know of genes implicated in the causation of familial/monogenic migraine, this information may have a role to play when prioritizing suggestive candidate genes in future migraine GWASs. Our knowledge about familial/monogenic migraine (e.g., hallmarks of less prevalent migraine types) can potentially help us to better understand underlying causes of common migraine. One question worth answering is “*does common migraine share genetic risk factors with familial/monogenic migraine?”*. Recent evidence points to some degree of shared genetic risk factors (55).

When using gene prioritization approaches, one needs to pay attention to limitations. For example, there may be a difference in prioritization performance between monogenic and polygenic disorders (focusing on predicting novel disease genes) (56), potentially due to “*the assumption of functional coherence among genes contributing to the same disease”* and the fact that “*complex diseases tend to perturb multiple biological processes”*, as stated by Linghu et al. (56). Moreover, the choice of gene prioritization tool(s) and the combination of gene prioritization components (evidence sources, prioritization strategy and input requirements) is key to enhance accuracy and precision. So, how do you best separate signal from noise?

The proposed gene prioritization approach is likely to be relevant for other fields, and could be used beyond that of causation. For example, the gene prioritization could be conducted from the perspective of disorder chronification or treatment effectiveness which then will guide the creation of training data (i.e., genes used to prioritize).

## Author Contributions

SF conceptualized the opinion piece and wrote the manuscript.

## Conflict of Interest

The author declares that the research was conducted in the absence of any commercial or financial relationships that could be construed as a potential conflict of interest.

## Publisher's Note

All claims expressed in this article are solely those of the authors and do not necessarily represent those of their affiliated organizations, or those of the publisher, the editors and the reviewers. Any product that may be evaluated in this article, or claim that may be made by its manufacturer, is not guaranteed or endorsed by the publisher.
